# Corneal Transduction by Intra-Stromal Injection of AAV Vectors *In Vivo* in the Mouse and *Ex Vivo* in Human Explants

**DOI:** 10.1371/journal.pone.0035318

**Published:** 2012-04-16

**Authors:** Claire Hippert, Sandy Ibanes, Nicolas Serratrice, Franck Court, François Malecaze, Eric J. Kremer, Vasiliki Kalatzis

**Affiliations:** 1 Institut de Génétique Moléculaire de Montpellier, CNRS, Montpellier, France; 2 Universités Montpellier I & II, Montpellier, France; 3 Inserm U563, Toulouse, France; 4 Département d'Ophtalmologie, Hôpital Purpan, Toulouse, France; Innsbruck Medical University, Austria

## Abstract

The cornea is a transparent, avascular tissue that acts as the major refractive surface of the eye. Corneal transparency, assured by the inner stroma, is vital for this role. Disruption in stromal transparency can occur in some inherited or acquired diseases. As a consequence, light entering the eye is blocked or distorted, leading to decreased visual acuity. Possible treatment for restoring transparency could be via viral-based gene therapy. The stroma is particularly amenable to this strategy due to its immunoprivileged nature and low turnover rate. We assayed the potential of AAV vectors to transduce keratocytes following intra-stromal injection *in vivo* in the mouse cornea and *ex vivo* in human explants. In murine and human corneas, we transduced the entire stroma using a single injection, preferentially targeted keratocytes and achieved long-term gene transfer (up to 17 months *in vivo* in mice). Of the serotypes tested, AAV2/8 was the most promising for gene transfer in both mouse and man. Furthermore, transgene expression could be transiently increased following aggression to the cornea.

## Introduction

The cornea is the transparent, avascular tissue at the front of the eye that covers the iris. As well as acting as a protective barrier to physical and pathogenic injury, the cornea is the major refractive surface of the eye [1]. It is primarily composed of three zones: an external stratified epithelium, a thick collagenous stroma, and a cuboidal monolayer of epithelial-like cells called endothelium. On the anterior side, the corneal epithelium consists of 6–7 layers of cells separated by tight junctions. The epithelium has a regenerative turnover of 1–2 weeks. On the posterior side, the endothelium is a non-regenerative monolayer of cells, which forms a leaky barrier regulating the hydration of the cornea. In the centre of the cornea is the stroma, which is primarily composed of an extracellular matrix and makes up 90% of the corneal thickness. The predominant residing stromal cell type is the keratocyte, a type of specialised fibroblast, which plays a role in general repair and maintenance [2].

Disruption of corneal transparency due to disease, infection or injury, results in the blocking or distortion of light entering the eye and hence leads to decreased visual acuity. Corneal pathologies are a significant and underestimated cause of unilateral blindness, leading to between 1.5 and 2 million new cases each year (http://www.who.int/blindness/causes/priority/en/index9.html). In the most severe cases, corneal transplantation is required. However, despite the fact that the cornea is an immunoprivileged organ, graft rejection is relatively common and thus local immunosuppressive treatments are required for successful grafting. With regards to the long-term outcome, transplanted cornea have a relatively limited lifespan, usually due to new injury, low level chronic rejection or progression of initial illness [3]. In light of these caveats, accessibility of the cornea renders viral-based gene therapy a viable alternative in the case of certain inherited and acquired diseases [4]. The outermost epithelium is the most accessible, however, paradoxally, gene transfer to this tissue has proven the most challenging due to its impenetrability and high turnover [5], [6]. In contrast, gene transfer to the innermost endothelium is feasible following injection of viral vectors into the anterior chamber [7], [8], [9], [10], with which it is in direct contact.

The corneal stroma plays the most important role in corneal transparency [1]. This tissue is separated from the corneal epithelium by a condensed collagenous layer, Bowman's membrane, and from the endothelium by a thin acellular layer, Descemet's membrane. Owing to this isolation, previous studies have shown that administration of viral vectors via the epithelium or endothelium does not result in efficient transduction of the stromal keratocytes [1]. Techniques allowing direct access of vectors to the stroma include topical application following lamellar keratotomy and laser (or mechanic) ablation of the epithelium. Although these routes of administration result in stromal transduction, transgene expression is generally limited to the anterior surface of the cornea in the vicinity of the lesion [9], [11], [12], [13], [14]. In contrast, in 2004, Carlson *et al.* reported efficient and widespread transduction of the stroma following direct injection [15], thus suggesting the potential of this technique for clinical application.

In addition to route of administration, another factor for efficient transduction is the choice of viral vector. Various studies have addressed delivery into the cornea with retroviral, lentiviral, herpes simplex and adenovirus vectors, with varying efficiencies depending on the cells targeted (for review see [1], [5]). More recently, attention has focused on the use of adeno-associated viral (AAV) vectors for ocular gene therapy, likely due to the proof of principal of these vectors for the treatment of retinal degeneration in humans [16], [17]. *In vivo* studies, in preclinical models, have demonstrated stromal transduction with AAV vectors following removal of the epithelium, and efficiency depended on the serotype tested [12], [14].

In this study, we targeted the corneal stromal cells by direct injection. We compared four AAV serotypes for their transduction efficiency and duration *in vivo* in the mouse cornea, and *ex vivo* in human corneal explants. We found that, in murine and human corneas, we transduced cells throughout the stroma via a single injection, preferentially targeted keratocytes, and achieved long-term gene transfer. Of the four serotypes, AAV2/8 was the most efficient in mice and humans. Furthermore, transgene expression could be reactivated when the stroma was damaged.

## Results

### I. *In vivo* mouse cornea studies

#### AAV2/8 transduction is the most efficient

**Figure 1:**
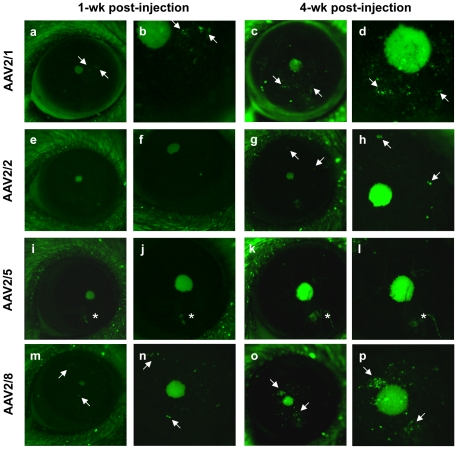
Transduction efficiency of AAV vectors in the mouse cornea. EGFP expression (indicated by arrows) in the mouse cornea detected by *in vivo* epifluorescence microscopy 1-wk post-injection of AAV2/1 (**a**), AAV2/2 (**e**), AAV2/5 (**i**) and AAV2/8 (**m**) vectors. (**b**, **f**, **j**, **n**) Higher magnification of **a**, **e**, **i** and **m**, respectively. (**c**, **g**, **k**, **o**) EGFP expression in the same corneas detected 4-wk post-injection. (**d**, **h**, **l**, **p**) Higher magnification of **c**, **g**, **k** and **o**, respectively. Magnifications **a**, **e**, **g**, **i**, **k**, **m**, **o**: 20×; **b**: 43×; **c**: 25×, **d**: 53×; **f**, **j**: 35×; **h**: 44×, **l**: 33×; **n**: 40×; **p**: 45×. For reference, the diameter of an adult mouse eye is ∼3.5 mm. The large green spot in centre of photos is the pupil of the mouse eye. The asterisk in panels **i** to **l** indicates an opaque lesion on the mouse eye that was present from the beginning of the experiments.

We produced four AAV vectors harbouring the AAV2 inverted terminal repeats (ITRs) and capsids from serotypes 1, 2, 5 or 8. Each vector contained an enhanced green fluorescent protein (EGFP) transgene under control of a cytomegalovirus (CMV) early promoter. To compare the onset and duration of expression of the four serotypes, we administered the same number of vector genomes (vg; dictated by the maximum volume (2 µl) injectable) of each vector into the eyes of two mice (*i.e.* 2 mice per vector) by intra-stromal injection. EGFP fluorescence was assayed *in vivo* once a week for a month using a fluorescence stereomicroscope adapted for small animal imaging. One-week post-administration, EGFP fluorescence was detected in the eyes injected with AAV2/1 (Fig. 1a,b) and AAV2/8 (Fig. 1m,n), and epifluorescence continued to increase over one month (Figs. 1c,d and 1o,p, respectively). Generally, the EGFP signal was stronger with the AAV2/8 vector. From 4-wk post-injection, fluorescence was detected in the mouse eyes injected with AAV2/2 (Figs. 1e–h), although at a relatively low level. In 3 of 4 eyes, we did not detect EGFP fluorescence from AAV2/5 (Figs. 1i–l); in 1 eye a transient signal appeared at week 2 that disappeared by week 3.

Taken together, our results suggested that AAV2/8 was the most efficient serotype for transducing the murine corneal stroma.

#### AAV2/8 transduction continues at least 17 mo *in vivo*

**Figure 2:**
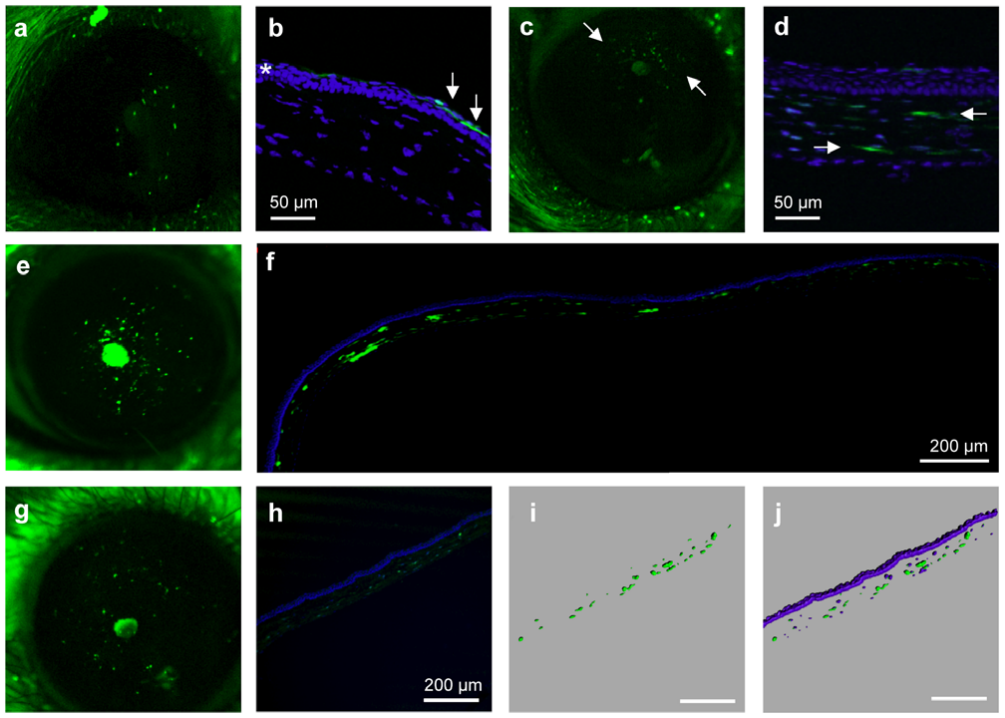
Onset and duration of AAV2/8 transgene expression. (**a**) Fluorescence detected by *in vivo* microscopy 3-d post-injection of AAV2/8. (**b**) At this time-point, EGFP expression (arrow) was in the corneal epithelium (asterisk) as determined by histological epifluorescence studies. EGFP (arrows) could be detected in the stroma by 1-mo post-injection by *in vivo* (**c**) and histological (**d**) studies. EGFP expression continued throughout the mouse cornea at 6-mo post-injection *in vivo* (**e**) and by histological studies (**f**; montage of two overlapping photographs). EGFP expression persisted at 17-mo post-injection (longest time-point tested) as seen by *in vivo* (**g**) and histological studies (**h**). (**i**, **j**) Processing and analysis of the stack acquisition of the 10-µm-thick section shown in panel **h** using the Imaris software. Magnifications **a**, **c**, **e** and **g**: 20×.

Following intra-stromal injection, EGFP expression from AAV2/8 was apparent at 3-d post-injection (Fig. 2a). Transgene expression was localised to the corneal epithelium by histological studies (Fig. 2b). This relatively short-lived signal was likely due to vector leaking into the epithelium because of the needle's trajectory. From 7-d post-injection, a signal in a different focal plane became apparent by *in vivo* microscopy. These latter EGFP+ cells were clearly visible by day 35 (Fig. 2c) and were located in the stroma by histological studies (Fig. 2d). At 6-mo post-injection, the EGFP-expressing cells could still be seen (Fig. 2e) throughout the stroma (Fig. 2f) and fluorescence continued to be detected up to 17-mo post-injection, the longest time point studied (Figs. 2g–j).

These results demonstrated that transgene expression from the AAV2/8 vector was long-lived (≥17 mo) in the corneal stroma. However, although a dose-dependent effect was observed (higher doses resulted in stronger, and more rapid onset of, expression), the initial number of EGFP-expressing cells was lower than that obtained with other vectors, notably adenoviruses ([15] and our data with human adenovirus serotype 5 (Ad5; Figure S1a–d) and canine adenovirus serotype 2 (CAV-2; Figure S1e–h)).

#### AAV2/8 transduces mouse keratocytes

**Figure 3:**
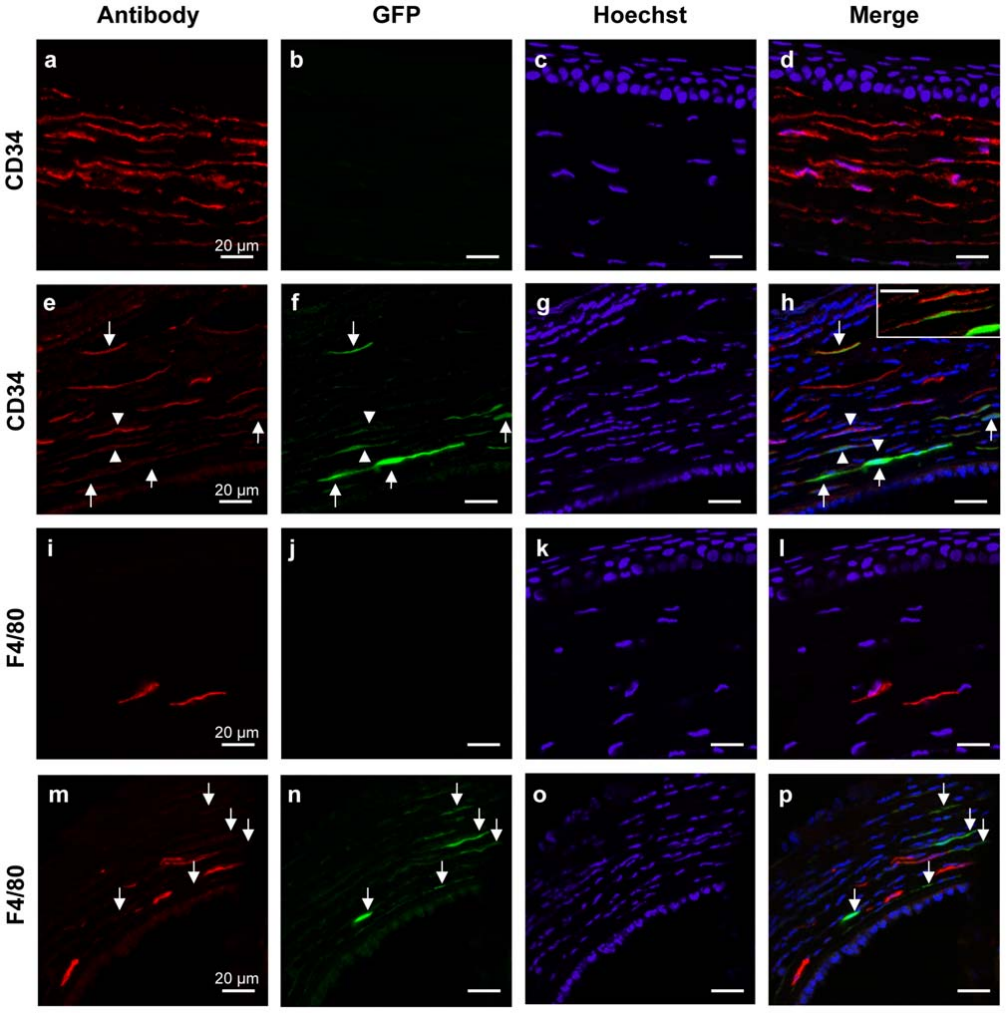
Cell specificity of AAV2/8 transduction. (**a**–**d**) Anti-CD34 staining (in red) of a non-injected mouse cornea shows abundant CD34+ cells. (**e**–**h**) Anti-CD34 labelling (in red) of a mouse cornea 24 h post-injection with 3×10^9^ vg of AAV2/8. Intra-stromal injection results in a decrease in the proportion of CD34+ cells (in red) due to an augmentation in the number of cell nuclei (in blue). The EGFP signal co-localises (arrows and arrowheads) with the CD34-labelled cells. Inset in **h** shows a higher magnification of the cells indicated by arrowheads minus the Hoechst filter. (**i**–**l**) Anti-F4/80 staining (in red) of a non-injected mouse cornea showing a low number of F4/80+ cells. (**m**–**p**) Anti-F4/80 labelling (in red) of a mouse cornea 24 h post-injection with 3×10^9^ vg of AAV2/8. The EGFP signal does not co-localise (arrows) with the F4/80-labelled cells.

In mice and humans, keratocytes have characteristic interconnecting dendritic processes and constitute the major (96%) cell type of the cornea [2]. The number and morphology of the EGFP-expressing cells observed under the microscope was consistent with keratocytes. There exist a number of established markers that can be used to identify human keratocytes, whereas this is not the case for the mouse cells. A widely used marker for human keratocytes is CD34 [18]. By contrast, in the mouse stroma, CD34 was reported as a marker of hemopoietic stem cells that have a rounded shape and represent ∼2.5% of the stromal population [19]. In the absence of a commercially available mouse keratocyte antibody, we incubated sections with an anti-CD34 antibody. In the control stroma (Figs. 3a–d), the CD34+ cell population represented approximately 100% of the stromal cells, which was reminiscent of keratocytes. Consistently, the morphology of the CD34+ cells was elongated rather than rounded. In the AAV2/8-transduced cornea (Figs. 3e–h), the number of CD34+ cells was reduced to 17.83±2.3% (compare Fig. 3e with 3a). This decrease in the proportion of CD34+ cells appeared to be due to either cell proliferation or migration as suggested by the increased number of cell nuclei following injection (compare Fig. 3g with 3c). We detected 4.1±0.8% of EGFP+ cells in the transduced stroma and observed a co-localisation of the CD34 and EGFP signals (Fig. 3h).

The remaining 1.5% of the mouse stromal cells was reported to be macrophages [19], [20]. Therefore, we incubated 10-µm thick sections from control and AAV2/8-transduced mouse corneas with an antibody against the macrophage marker F4/80. In the control stroma (Figs. 3i–l), 18.8±4.9% of the cells were F4/80+. In the AAV2/8-transduced cornea (Figs. 3m–p), we detected 7.8±2% of F4/80+ cells (compare Fig. 3m with 3i) and an augmentation in the number of cell nuclei (compare Fig. 3o with 3k). Nonetheless, we did not observe co-localisation between the F4/80 and EGFP signals (Fig. 3h).

Taken together, we concluded that the AAV2/8 vector did not transduce resident stromal macrophages. Based on morphology and number, the transduced cells were consistent with keratocytes. Although we cannot formally rule out transduction of CD34+ hemopoetic stem cells, the number and shape of these cells were incompatible with this population.

#### AAV2/8 transgene expression can be hyper-activated *in vivo*

**Figure 4:**
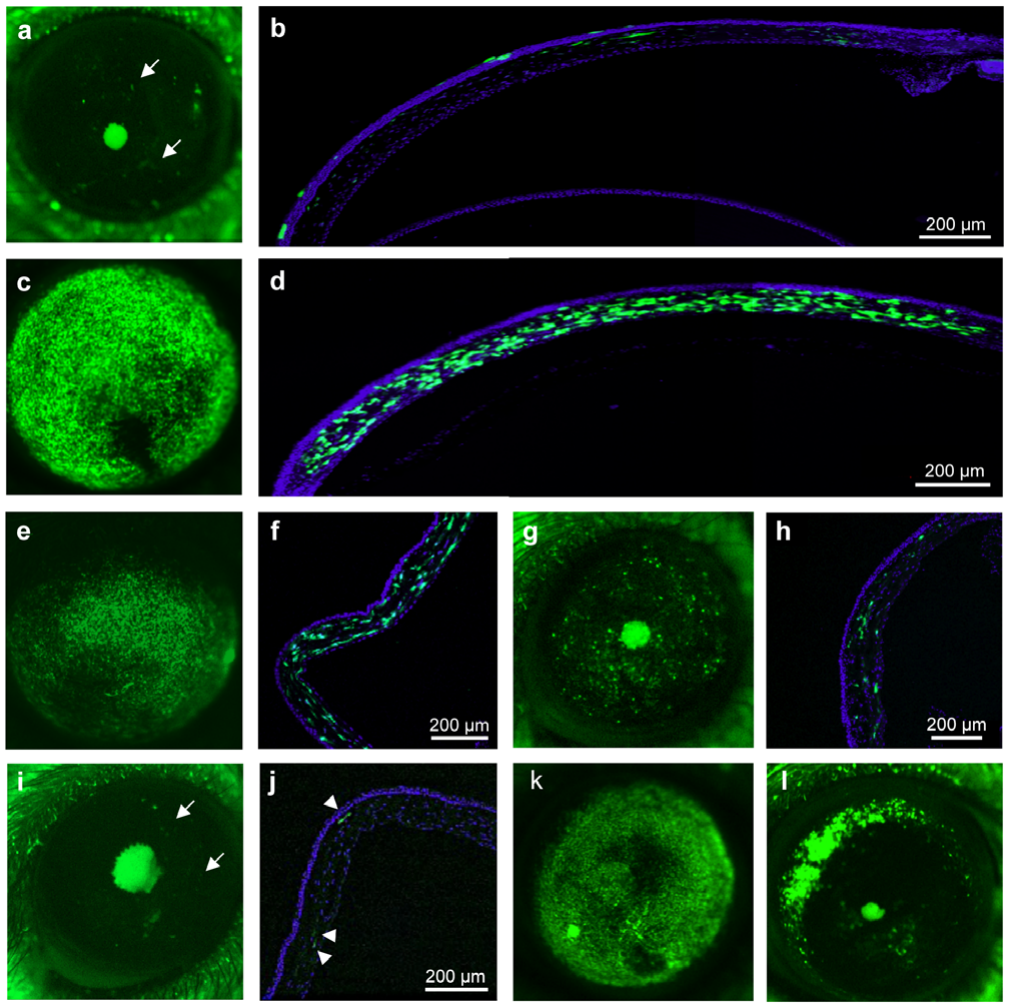
EGFP expression levels following PBS injection in the transduced mouse cornea. EGFP expression 7-d post-AAV2/8 injection by *in vivo* (**a**) and histological (**b**) studies. (**c**, **d**) Increase in EGFP expression 24 h after PBS injection of the AAV2/8-transduced cornea. Panels **b** and **d** are composed of two overlapping photographs. (**e**) Increase in EGFP expression from AAV2/1 (*cf.* Fig. 1c) following a second injection of PBS. (**f**) Histological section showing the localisation of EGFP expression from AAV2/1 to the corneal stroma. **g**) Smaller increase in EGFP expression from AAV2/2 (*cf.* Fig. 1g) following PBS injection. (**h**) Histological section showing the localisation of EGFP expression from AAV2/2 to the corneal stroma. (**i**) Barely detectable EGFP expression (arrows) from AAV2/5 (*cf.* Fig. 1k) following PBS injection. (**j**) Histological section showing the localisation of EGFP expression (arrowheads) from AAV2/5 to the corneal stroma. (**k**) Hyper-activation of EGFP expression in a mouse eye that was injected with PBS 94 d post-AAV2/8 injection. (**l**) Less extensive activation of EGFP expression in the mouse eye shown in panel **k** following a second PBS injection 117 d post-AAV2/8 injection. Magnifications **a**, **c**, **l**: 26×; **e**, **g**, **i**, **k**: 22×.

In contrast to most internal organs, the cornea is continuously exposed to environmental aggressions and pathogens. In particular, some adenovirus serotypes are common etiologic agents for external ocular infection [21], [22]. To evaluate the effect of transient aggression subsequent to AAV delivery, we injected the AAV2/8-transduced mouse cornea with a first generation human Ad5 vector expressing beta-galactosidase (Adβgal; [23]), with lipopolysaccharide (LPS), or with PBS 1-wk post-AAV administration, and recorded EGFP levels 24 h later (adenovirus vectors and LPS, but not PBS, are known inflammatory agents). As mentioned above, at 1-wk post-AAV2/8 administration, we began to see EGFP expression in the corneal stroma (Figs. 4a,b). When we re-injected the corneas with Adβgal, LPS or PBS at this time-point and assayed *in vivo* fluorescence 24 h later, we detected a significant hyper-activation in EGFP expression throughout the corneal stroma (Figs. 4c,d). The expression was not detectable 1 wk later (data not shown). To exclude an aggravation due to phosphate ions, as PBS was present in all three conditions, we performed a second injection with saline, which increased the number of EGFP+ cells to the same extent (data not shown). Notably, EGFP expression was boosted in the corneal stroma regardless of AAV serotype, but the intensity of the boost was serotype-dependent: AAV2/8 (Figs. 4c,d)>AAV2/1 (Figs. 4e,f)>AAV2/2 (Figs. 4g,h)>AAV2/5 (Figs. 4i,j). Furthermore, EGFP expression from AAV2/8 could be reactivated by PBS injection up to 94 days (latest time point tested) after the initial AAV2/8 injection (Fig. 4k). We also tested whether we could repeatedly reactivate EGFP expression by reinjecting the same eye a second time with PBS (on day 117 post-AAV2/8 injection). EGFP expression was reactivated but less extensively (Fig. 4l).

**Figure 5:**
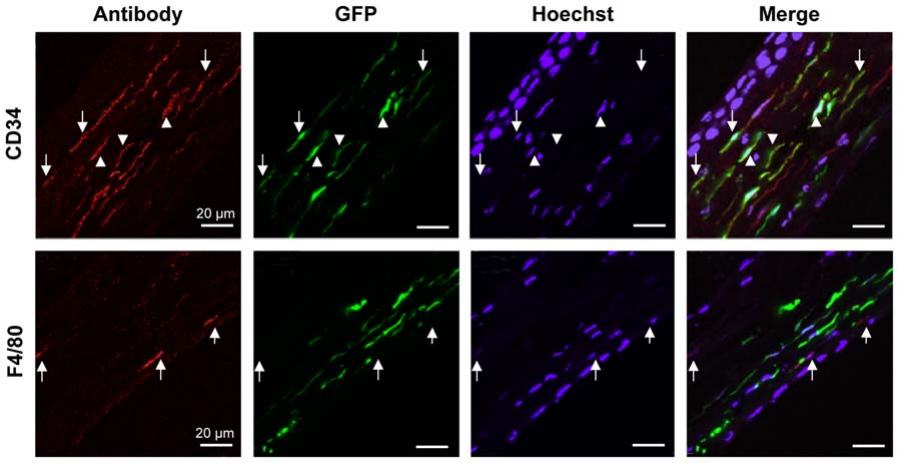
EGFP-expressing cell population following PBS injection in the transduced mouse cornea. (**a**–**d**) Anti-CD34 staining (in red) of a mouse cornea 24 h post-PBS injection (same eye shown in Fig. 4C). The CD34 signal co-localises with the EGFP signal (arrows and arrowheads). (**e**–**f**) Anti-F4/80 staining (in red) of a mouse cornea 24 h post-PBS injection. The F4/80 signal does not co-localise with the EGFP signal (arrows).

To identify the EGFP-expressing cells following PBS injection, we incubated sections of a PBS-injected mouse eye with an anti-CD34 antibody. We clearly identified 33±8.5% of CD34+ cells in the stroma; counting was difficult due to the faint red signal coupled to the elongated shape of the cells and, as a consequence, the percentage of CD34+ cells was likely underestimated. We observed 57.8±5.7% of EGFP+ cells, and the EGFP signal co-localised with the CD34 signal (Figs. 5a–d). Following incubation with an anti-F4/80 antibody, we detected 8.4±0.7% of F4/80+ cells. By contrast, we did not observe a co-localisation of the F4/80 and EGFP signals (Figs. 5e–h). These observations indicate that the CD34+/F4/80− population showing hyper-activated EGFP expression is the same cell population as that initially transduced by AAV2/8 administration.

Taken together, these results suggested that an aggression of the cornea caused a transient boost in EGFP expression. Furthermore, the intensity of the boost was relative to the initial efficiency of transduction of the serotype used. This trend suggested that the fold increase per serotype was similar. The commonality with each vector is of course the AAV2 genome, which suggests that a cellular response to aggression can induce transcription from the ITRs and/or the CMV promoter.

#### AAV2/8 vector genomes decrease while mRNA levels increase after corneal re-injection

**Figure 6:**
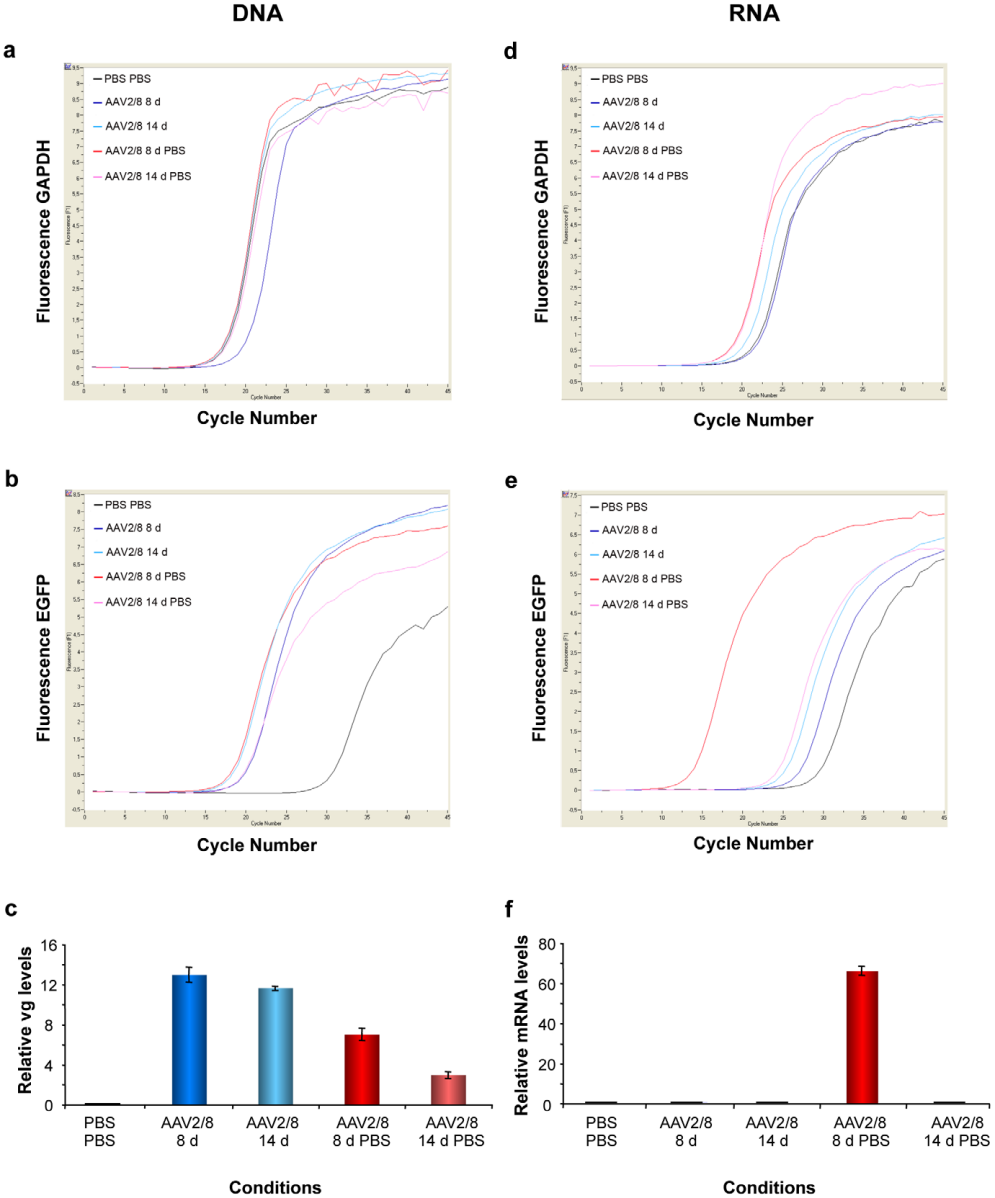
Real-Time PCR analysis of vector genome (a,b,c) and mRNA (d,e,f) levels following AAV2/8 transduction of mouse cornea. Amplification curves of (**a**) GAPDH and (**b**) EGFP in DNA extracted from injected mouse eyes. To aid visualisation, only one of the duplicate curves is shown. (**c**) Graphical representation of the concentration of EGFP normalised to that of GAPDH in each sample (the colour of the curves in **a and b** matches those of the corresponding bars in **c**). As a control, EGFP DNA could not be detected following injection and re-injection of the mouse cornea with PBS (PBS PBS). In contrast, EGFP DNA levels were detected and relatively stable both 8- and 14-d post-AAV2/8 transduction (blue bars). Following a second injection of PBS 7-d post-transduction, a 1.9- and 4-fold decrease in EGFP DNA was detected on days 8 and 14, respectively (red bars). Amplification curves of (**d**) GAPDH and (**e**) EGFP in RNA extracted from injected mouse eyes. (**f**) Graphical representation of the concentration of EGFP normalised to that of GAPDH in each sample (the colour of the curves in **d and e** matches those of the corresponding bars in **f**). As a control, EGFP RNA levels could not be detected following injection and re-injection of the mouse cornea with PBS. Similarly, EGFP RNA levels could not be detected 8- and 14-d post AAV2/8 transduction. In contrast, a second injection of PBS 7-d post-transduction resulted in a 65-fold increase in EGFP RNA levels on day 8 (red bar). RNA levels were no longer detectable by day 14.

To determine whether the rapid decline in EGFP expression following a second corneal injection was due to the loss of vector genomes or a return to the previous level of transcription, we followed the fate of AAV2/8 DNA (Figs. 6a,b,c) and mRNA (Figs. 6d,e,f) using Real-Time PCR. We found that vector genome levels were relatively stable both 8- and 14-d post-AAV2/8 injection (Fig. 6c). In contrast, following injection with PBS at day 7 post-AAV2/8 injection, we detected a 1.9-fold decrease at day 8, and a 4-fold decrease at day 14.

An analysis of EGFP mRNA levels showed that levels were at background on days 8 and 14 post-AAV2/8 administration in the absence of a second injection of PBS (Fig. 6f). In contrast, PBS injection on day 7 resulted in a 65-fold increase in EGFP mRNA levels on day 8, which returned to background levels by day 14. This kinetics of mRNA levels was consistent with that of EGFP expression observed by fluorescent microscopy.

Taken together, these results indicated that a second injection in the cornea caused a partial loss in vector genomes concomitant with a transient increase in mRNA levels. The subsequent reduction of EGFP expression appeared to be due to the return to basal levels of vector mRNA.

### II. *Ex vivo* human cornea studies

#### AAV2/8 efficiently transduces human corneas and preferentially targets keratocytes

**Figure 7:**
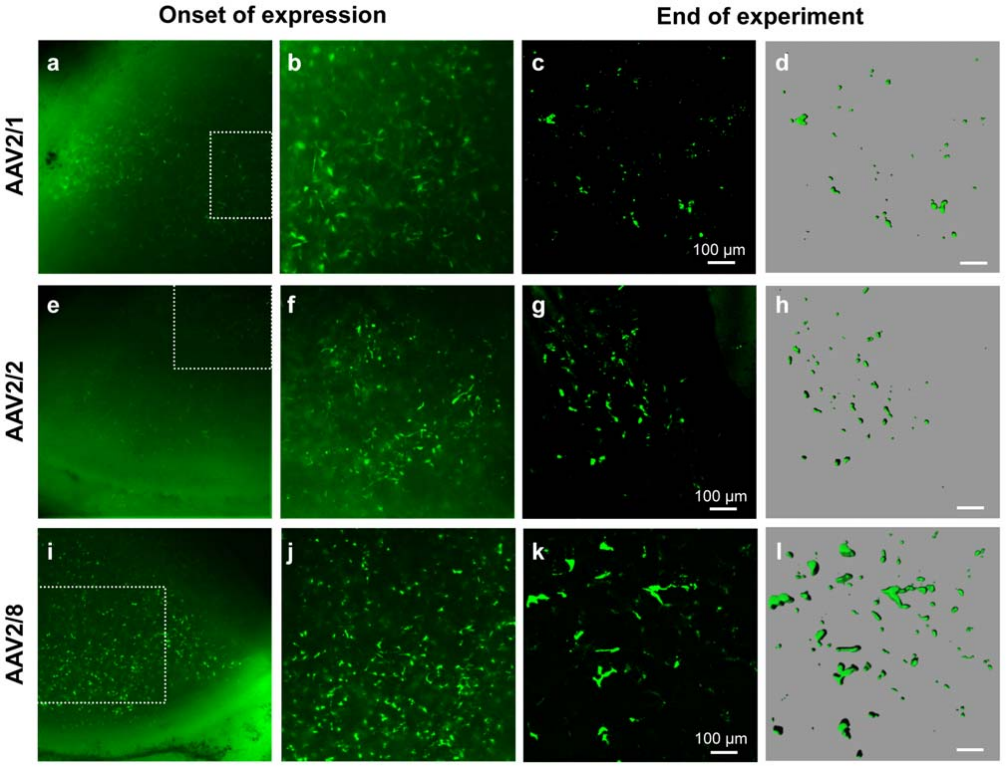
Transduction efficiency of the AAV vectors in the human corneal explants. One-wk post-injection, using *in vivo* microscopy, EGFP expression can be seen in throughout the cornea following intra-stromal injection of the vectors AAV2/1 (**a**) and AAV2/8 (**i**). (**b**, **j**) Higher magnification of the boxed regions in **a** and **i**, respectively. (**e**) Three-weeks post transduction, EGFP expression can be detected following AAV2/2 injection. (**f**) Higher magnification of the boxed area in **e**. (**c**, **g**, **k**) EGFP expression on histological sections of each cornea 8-wk post-injection of AAV2/1, −/2, −/8, respectively. (**d**, **h**, **l**) Imaris-treated images of **c**, **g**, **k**, respectively, showing EGFP-expressing cells.

To be clinically relevant, we evaluated AAV vector transduction efficiency in human corneal explants. We injected each vector into the stroma and EGFP expression was followed weekly by i*n vivo* fluorescence microscopy. One-week post-injection, fluorescence could be detected in the human corneas injected with AAV2/1 (Fig. 7a,b) and AAV2/8 (Fig. 7i,j), although expression was higher with the AAV2/8 vector. From 3-wk post-injection, fluorescence was detected in the corneas injected with AAV2/2 (Figs. 7e,f), although at a relatively low level. Over the duration of the experiment, we did not detect a significant signal following injection with AAV2/5 (data not shown). For the AAV2/1, 2/2 and 2/8 vectors, expression persisted for ∼8 wk (endpoint of experiment), however, the qualitative and quantitative expression profiles varied: AAV2/8-transduced cells expressed high levels of GFP, and their size (80–200 µm; [24]) and shape was consistent with keratocytes (Figs. 7k,l). By contrast, the EGFP-expressing cells transduced with the AAV2/1 (Figs. 7c,d) or −/2 (Figs. 7g,h) vectors were smaller, less abundant, and did not display a characteristic dendritic form.

**Figure 8:**
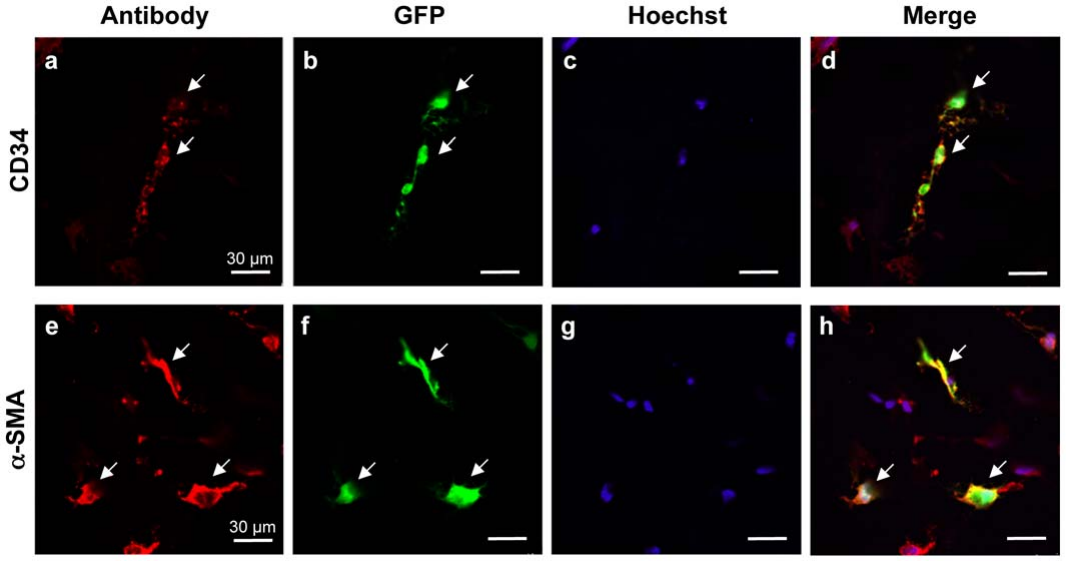
Intra-stromal injection of AAV2/8 in the human cornea results in transduction of keratocytes. (**a–d**) Anti-CD34 labelling (in red) of a human cornea co-localises with EGFP-expressing cells (in green) and identifies these cells as quiescent keratocytes. (**e–h**) Anti-α–SMA labelling (in red) of a human cornea co-localises with EGFP-expressing cells (in green) and identifies these cells as activated keratocytes.

As mentioned above, CD34 is a marker for quiescent human keratocytes [18], [25]. Once keratocytes become activated and differentiate into myofibroblasts, they stop expressing CD34 and begin expressing alpha-smooth muscle actin (α-SMA; [26]). Consequently, we incubated 10-µm-thick sections from AAV2/8-transduced human corneas with antibodies against these markers. Co-localisation of the EGFP signal with anti-CD34 (Figs. 8a–d) and anti- α-SMA (Figs. 8e–h) labelling indicated that the transduced cells were both quiescent and activated keratocytes.

Taken together, these results showed that the transduction efficiency of the AAV vectors was similar between the mouse and human corneas, and that AAV2/8 was the most efficient serotype for transducing the corneal keratocytes of both species.

## Discussion

The eye is highly amenable to gene therapy because it is i) accessible, ii) small and enclosed allowing the use of low vector doses, and iii) sequestrated from the general circulation rendering it immune-privileged. The retina has received the most attention in recent years culminating in the successful clinical trials in 2008 for Leber's congenital amaurosis, a hereditary congenital blindness due to a breakdown of the visual cycle [16], [17]. An AAV2/2 vector was used to deliver the missing gene into the retinal pigment epithelium allowing an amelioration/restoration of sight in visually impaired subjects. Thus, at least for now, AAV vectors appear to be safe and efficient for the eye.

Although the field is less advanced, there is a growing interest in using gene therapy to target the cornea. Potential applications are the correction of corneal neovascularisation [10], [27] or scarring [28], [29], as well as the treatment of certain genetic diseases [4]. Among these, the multisystemic lysosomal storage diseases (LSDs) are particularly appealing candidates as the associated corneal symptoms are not alleviated by systemic treatment in contrast to other organs [30]. Corneal anomalies in LSDs often include corneal clouding (as in the case of mucopolysaccaridosis (MPS) type VI and VII; [4]) or photophobia (as in the case of cystinosis; [31], [32]) and are due to abnormal storage in the stromal keratocytes.

A highly efficient way to transduce stromal keratocytes is to administer vectors via intra-stromal injection. This technique causes a transient separation of the stromal matrix allowing the injected liquid to be distributed throughout. In this way, we achieved widespread transduction of the stroma, in contrast to topical administration following lamellar keratotomy [9], [11] or laser ablation [12], [13] of the epithelium, which result in local transduction. Furthermore, as the stroma is delineated by Bowman's and Descemet's membranes, vector dissemination and transduction of the epithelium or endothelium appeared negligible. Within 24 h following injection, the cornea returned to its pre-injected transparent state, likely due to the removal of the injected liquid to the external medium by the pump activity of the endothelial cells. Underlying this transformation is also the highly efficient and unique repair mechanism intrinsic to the cornea [33], which rapidly restores transparency. However, this repair mechanism also represents a challenge for achieving long-term gene expression. For example, in 2004, Carlson *et al.* demonstrated the potential of intra-stromal injection by administrating a human adenovirus vector containing a transgene under control of the keratocan promoter [15]. Consistent with our observations with adenovirus vectors, transduction was throughout the cornea, but peaked at 24 h and then declined significantly by 1 wk. Although the corneal keratocytes are quiescent cells, and thus an ideal population for non-integrative vectors, they have the particularity of losing their quiescence and differentiating into activated myofibroblasts during corneal repair. In terms of timing, keratocytes immediately adjacent to the lesion begin to undergo cell death within a few hours after injury creating an acellular zone [33]. Six hours post-injury, keratocytes neighbouring this zone begin to lose their quiescence, become activated, divide, and within 24 h migrate towards the damaged area. Repopulation begins from around 48 h. This cell death and division process is consistent with clearance of the transduced cells and loss of the episomal vector genomes, respectively.

**Figure 9:**
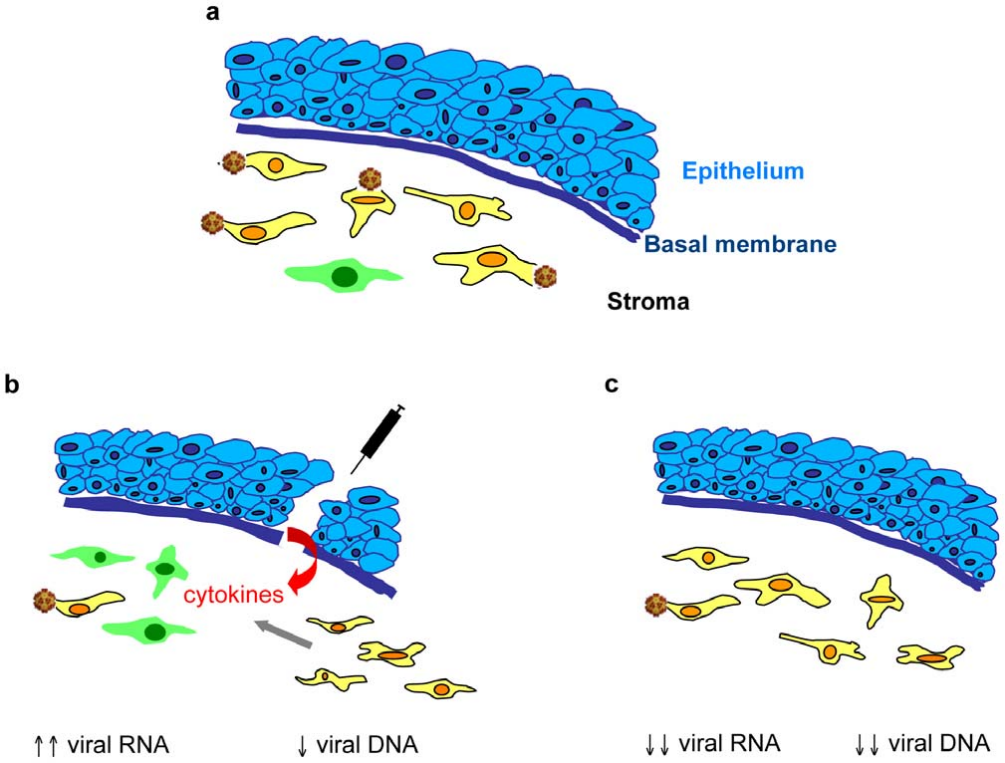
Schematic representation of the events following PBS injection and hyper-activation of EGFP expression. (**a**) Following initial AAV2/8 injection, a large number of cells harbour viral particles or vector genomes but a small number of cells express EGFP (in green). (**b**) Twenty-four h post-PBS injection, the disruption of the epithelial basement membrane results in the release of cytokines that induce the repair process. This process involves cell death in the stroma immediately underlying the injection site, which reduces the vector genome levels (∼2-fold decrease), and cell migration from the limbal region. The ensuing events also result in either *de novo* uncoating of encapsidated virions or reactivation of transcriptionally-silenced genomes leading to activated EGFP expression (consistent with the 65-fold increase in EGFP mRNA levels). (**c**) One-week post-PBS injection, the cornea has returned to its basal state and is no longer expressing EGFP (low mRNA levels). The cell death process has removed a number of transduced keratocytes (4-fold decrease in vector genome levels). However, cells harbouring the virions are still present (persisting DNA levels) as a subsequent PBS injection still can activate EGFP expression but less extensively.

By contrast, we found that, in general, transgene expression from AAV vectors was detected from 1 wk post-injection (earlier at higher doses) and increased during the following weeks, likely due to the lag time required for their single stranded genome to convert to double-stranded. Moreover, transgene expression was still detectable at 17-mo post-administration. AAV, like Ad, vectors are theoretically “non-integrating" and should follow the same fate after corneal repair. It is possible that i) AAV vectors integrate into stromal keratocytes [34], ii) the episomal vector genomes form long concatamers that are not lost during cell division [35], or iii) some of the vector genomes remain encapsidated in the cell [36] and retain infectivity [37], thus potentially entering neighbouring cells. Along this line, intact AAV particles were reported for up to 6 years following successful gene transfer and, interestingly, these particles were found in cells that did not express the transgene [36]. Regardless, a certain number of AAV-carrying cells are inevitably eliminated during the initial corneal repair mechanism. Thus, we hypothesise that the relatively lower numbers of transgene-expressing cells following AAV-transduction (versus Ad-transduction) likely stems from the smaller percentage of AAV-carrying cells that persist following repair (Fig. 9a).

Eyes are the targets of many environmental and pathological stimuli. Thus, we explored possible adverse effects associated with a transient aggression. In 2002, Tsai *et al.* reported that transient ocular anterior segment inflammation provoked transgene expression in endothelial cells following injection of an AAV2/2 vector expressing beta-galactosidase from a CMV promoter into the anterior chamber of rabbits [38]. Inflammation was induced by LPS treatment whereas there was no effect following PBS injection. In our study, the intra-stromal administration of LPS (or Ad5) following AAV2 vector injection also provoked transgene expression. This suggested that a larger number of cells harboured AAV genomes than was initially suspected based on transgene expression, but that these were either still encapsidated or transcriptionally inactive. In contrast to the report by Tsai *et al.*, a PBS (or saline) injection also provoked transgene expression in our study. This suggests that in the case of intra-stromal administration, it is the lesion rather than the substance that causes the inflammation. More specifically, disruption of the basement epithelial membrane may result in the production of cytokines from the epithelium that induce the repair process: IL-1α initiates cell death in the underlying stroma and TGFβ transforms adjacent keratocytes into activated myofibroblasts (for review see [2]; Fig. 9b). We attempted to prevent inflammation by applying anti-inflammatory drugs (dexamethasone, sulfasalazine) prior to PBS injection, but the results were inconclusive.

Tsai *et al.* also reported that induced AAV-transgene expression in the endothelium gradually diminished as the inflammation subsided (by 50% 5-d post-injection and to background levels 15-d post-injection when inflammation was no longer detectable) [38]. In the case of the corneal stroma, transgene expression diminished more rapidly and was undetectable 1-wk post-injection. This is reminiscent of the timing of clearance following direct adenovirus administration [15]. Our Real-Time PCR data showed that the loss of transgene expression coincided with a decrease in vector genomes and a loss of mRNA. Consistent with the report by Tsai *et al.*, we also reactivated transgene expression with a subsequent (*i.e.* third) injection, albeit at lower levels. This transcriptional upregulation confirms that AAV genomes remained following the second wave of corneal repair (Fig. 9c), as suggested by the Real-Time PCR results at 14-d post-PBS injection. Based on our data, we cannot determine whether the reactivated transgene expression is due to *de novo* uncoating of encapsidated virions or reactivation of transcriptionally-silenced genomes.

*De novo* uncoating would be in harmony with the report by Johnson and Samulski showing that a population of AAV2 virions enter the nucleus following cell entry and accumulate in the nucleolus [37]. These virions may then use nucleolar disruption during mitosis, genotoxic stress or coinfection, as a trigger to release their genetic contents. Once released, the genome would need to undergo second strand synthesis, which is reportedly facilitated by DNA repair [39]. Inflammation leads to DNA damage of the host cell genome and results in active production of DNA repair enzyme and cofactor [38]. This would account for the immediately high level of transgene expression we observed following injection and disruption of the basement epithelial membrane (Fig. 9b). Furthermore, as non-disrupted virions released from the nucleolus retain infectivity [37], a proportion of them may be released and infect neighbouring cells following division of the mother cell. This could contribute to the lower level of transgene expression observed following another round of inflammation.

Intra-stromal injection is a viable technique for specifically transducing keratocytes throughout the corneal stroma. Moreover, AAV vectors are capable of long-term gene expression, although, the number of transduced cells that remain following injection and repair may not be sufficient to administer adequate amounts of a therapeutic protein in view of treating genetic diseases. The exception may be the case of some LSDs. For example, for LSDs due to a defective lysosomal enzyme such as in MPS VI or VII, the phenomena of cross-correction, whereby the lysosomal enzymes escape from cells and are taken up by neighbouring cells [40], requires gene transfer only to a subset of cells. Therefore, it is possible that AAV transduction could result in phenotypic correction. Alternatively, in the case of a non-secreted protein, such as a membrane transporter as is the case for cystinosis [41], our unpublished data indicate that the number of persisting, transduced keratocytes is insufficient to achieve correction via transgene expression. By contrast, Galiacy *et al.* recently addressed the potential of AAV gene therapy for treating or preventing corneal scarring, which can lead to corneal blindness. A single intra-stromal injection of an AAV2/8 vector encoding a fibril collagenase (MMP14), reduced the expression levels of major genes involved in scarring, suggesting a potential for therapeutic applications [29].

Is it possible to overcome/exploit the corneal repair mechanism to retain transduced cells and maintain activated transcription? One possibility would be to target keratocyte precursors residing in the stroma rather than keratocytes themselves. A resident population of stem cells has been identified in the adult human stroma [42]. These cells differentiate into keratocytes and restore transparency upon injection into the scarred corneas of mice [43]. Thus, if one could stably transduce this population *in vivo*, they could repopulate the cornea following repair. Along this line, the normal mouse cornea may contain two populations of bone marrow-derived leukocytes (totalling 4% of stromal cells), both of which are distinct from stromal keratocytes (96%): the larger population (∼2.5%) resembles CD34+ hemopoetic stem cells, whereas the smaller population (∼1.5%) are macrophages [19]. We observed transgene expression in CD34+ cells but not in F4/80+ cells. Therefore, if the transduced CD34+ cells do represent a stem cell population, it is tempting to speculate that these cells could repopulate the cornea following injury and differentiate into keratocytes.

Interestingly, the profile and efficiency of transduction of the different AAV serotypes in the cornea was consistent between mouse and man. Moreover, the transduced human cells were *bona fide* keratocytes. To our knowledge, this is the first study showing the transduction of human corneal explants by intra-stromal injection. Two previous reports detailed the transduction of human explants by incubation in a culture medium containing AAV vectors [12], [14]. In contrast to these studies, our approach results in widespread transduction of the stromal keratoyctes that was highest with AAV2/8, followed by AAV2/1 and that was low to negligible with AAV2/2 or −/5. Therefore, of the serotypes we tested, we identify AAV2/8 as the most potentially interesting for keratocyte transduction in the human cornea by intra-stromal injection.

## Materials and Methods

### Production of recombinant AAV vectors

AAV2 vectors harbouring the capsids from serotypes 1, 2, 5 and 8, and expressing EGFP, were produced by the Centre for Animal Biotechnology and Gene Therapy (CBATEG) at the Universitat Autonoma de Barcelona (Spain). The AAV-helper plasmids containing Rep2 and Cap for each serotype were kindly provided by JM Wilson, University of Pennsylvania, Philadelphia, PA, USA. Viral supernatants were concentrated on iodixanol gradients. The titres were: AAV2/1 – 5.3×10^11^ vg/ml; AAV2/2 – 1.9×10^12^ vg/ml; AAV2/5 – 8.8×10^11^ vg/ml; AAV2/8 – 1.56×10^12^ vg/ml.

### Ethics statement

All animal breeding and experiments were carried out in accordance with the European and National guidelines for the care and use of laboratory animals (Council Directive 86/6009/EEC). Institutional and regional ethics committees (“Comité d'éthique de la Languedoc Roussillon"; permit number CE LR 0709) reviewed and approved the work on animals. Post-mortem human corneal explants were collected by the Centre for Human Biological Collections of Montpellier (CCBH-M; CHRU Montpellier, France). Corneas that did not fulfil the endothelial density inclusion criteria for transplantation were provided for research purposes in accordance with French regulations by the tissue bank at the St Eloi hospital (CHRU Montpellier).

### *In vivo* transduction

C57BL/6 mice were maintained in a controlled environment with a 12 h/12 h light/dark cycle, housed in groups of 10 maximum and allowed food and water *ad libitum*. Prior to injection, animals were anaesthetised by an intraperitoneal injection of 10 mg/kg xylazine (Bayer Pharma, Puteaux, France) and 100 mg/kg ketamine (Merial, Lyon, France). Intra-stromal injection was performed by first creating a small incision in the corneal epithelium using the tip of a 26-gauge needle. The incision was performed equidistance between the corneo-scleral junction and the corneal centre. A 33-gauge needle attached to a 10 µl Hamilton microliter syringe (Sigma-Aldrich, St. Quentin Fallavier, France) was then introduced through the incision into the corneal stroma and 10^9^ vg of vector in 2 µl of PBS were injected (unless otherwise stated). For the secondary injections, 1-wk post-vector administration (unless otherwise stated), the previously-injected mouse cornea was reinjected with 2 µl of **a)** 10^9^ physical particles of Adβgal, **b)** 2 ng/µl LPS *Escherichia coli* 0127:B8 (Sigma Aldrich), **c)** PBS without Ca^2+^ and Mg^2+^ (Invitrogen, Cergy Pontoise, France) or **d)** saline (0.9% NaCl solution), and fluorescence observed 24 h and 1 wk later.

### Corneal explants and *ex vivo* transduction

Corneal explants were cultured in CorneaMax media (Eurobio, Courtaboeuf, France) at 31°C in sealed bottles. Intra-stromal injection was performed by inserting a 29-gauge needle attached to a 0.5 ml syringe through the endothelium into the stroma to administer a total of 5×10^10^ vg of AAV in 300 µl PBS.

### *In vivo* and *ex vivo* microscopy studies

Mice were anaesthetised using 2% isoflurane in an induction chamber with an 0_2_ flow rate of 0.2 L/min prior to observation. Fluorescence in the mouse eye and in the human corneal explants was observed using an M2Bio fluorescence (dissecting) stereomicroscope assembled by Kramer Scientific (Amesbury, MA, USA) on a Zeiss Stemi V6 stereomicroscope platform (Nanterre, France), and equipped with a CCD camera (Small Animal Imaging Platform (IPAM), Montpellier, France). Image acquisition was performed using the MetaMorph imaging program (Molecular devices, Wokingham, Berkshire, United Kingdom).

### Histological studies

Following sacrifice, mouse eyes were enucleated, fixed in 4% paraformaldehyde/PBS for 24 h, placed in 20% sucrose/PBS for 24 h, and embedded in OCT matrix (CellPath, Newton, Powys, UK). The same protocol was used for the human corneal explants. For the visualisation of EGFP fluorescence, the nuclei of 10-µm-thick sections were labelled with 0.2 µg/ml bisBenzimide Hoechst (Sigma-Aldrich) for 5 min prior to mounting in DakoCytomation fluorescent mounting media (Dako, Trappes, France). For the immunofluorescence studies, sections were blocked in 10% horse serum/2% BSA. Primary antibodies were incubated on sections for 1 h, and the secondary antibody incubated 45 min, at room temperature prior to Hoescht labelling and mounting. For the mouse sections, the primary antibodies used were 1∶300 dilution rat anti-mouse F4/80 (Clone BM8; Caltag laboratories, Invitrogen) and 1∶100 rat anti-mouse CD34 (Ebiosciences, Clinisciences, Montrouge, France) and the secondary antibody was 1∶1000 dilution chicken anti-rat IgG-Alexa Fluor 555 (Molecular probes, Invitrogen). For the human sections, the primary antibodies used were 1∶100 mouse anti-human CD34 (clone B1-3C5; Abcam, Cambridge, UK) and 1∶50 mouse anti-human α-SMA (Clone 1A4; R&D Systems, Lille, France) and the secondary antibody 1∶500 goat anti-mouse IgG-Alexa Fluor 546 (Molecular probes, Invitrogen). Sections were observed using a Zeiss LSM 510 META confocal microscope and image acquisition performed using MetaMorph. For a clear interpretation, certain images were further processed and analysed using the Imaris software (Bitplane Scientific Software, Zurich, Switzerland). Immunolabelled cells were quantified manually and the results expressed as the mean ± standard error of the mean (*n* = 3 to 11).

### Real-Time PCR amplification

Five groups, each comprising 6 mice, were used. **Group 1** (PBS PBS): Day 0 – PBS injected in one eye; day 7 - the injected eye was reinjected with 2 µl PBS; day 8 - the mice were sacrificed. **Group 2** (AAV2/8 8 d): Day 0 – AAV2/8 injected in one eye; day 8 - the mice were sacrificed. **Group 3** (AAV2/8 14 d): Day 0 – AAV2/8 injected in one eye; day 14 - the mice were sacrificed. **Group 4** (AAV2/8 8 d PBS): Day 0 – AAV2/8 injected in one eye; day 7 - the transduced eye was injected with 2 µl PBS; day 8 - the mice were sacrificed. **Group 5** (AAV2/8 14 d PBS): Day 0 – AAV2/8 injected in one eye; day 7 - the transduced eye was injected with 2 µl PBS; day 14 - the mice were sacrificed. Fluorescence was checked *in vivo* on day 8 (groups 1, 2 and 4) and on day 14 (groups 3 and 5). The transduced cornea of each mouse was divided in two, one half for DNA-, and the other for RNA-, extraction. The cornea was homogenised and DNA extracted by proteinase K digestion and phenol-chloroform extraction as previously described [44]. RNA was extracted individually from each cornea as described [45] and then, due to insufficient quantities, the RNA within each experimental group was pooled for cDNA synthesis. cDNA was synthesised from 1 µg total RNA using the Superscript III reverse transcriptase according to the manufacturer's instructions (Invitrogen, Cergy Pontoise, France). Genomic DNA and cDNAs were amplified by Real-Time PCR using a SYBR green mix [46] and a LightCycler apparatus (Roche Molecular Biochemicals) as described [47] The primers for the reaction were as follows: **EGFP F:** 5′ CAG AAG AAC GGC ATC AAG GT 3′; **EGFP R:** 5′ CTG GGT GCT CAG GTA GTG G 3′; **GAPDH F:** ACA GTC CAT GCC ATC ACT GCC 3′; **GAPDH R:** 5′ GCC TGC TTC ACC ACC TTC TTG 3′. EGFP and GAPDH concentrations were calculated using established standard curves and the results normalised by expressing EGFP concentration as a function of the GAPDH concentration per sample. Samples were assayed in duplicate.

## Supporting Information

Figure S1 **Transduction of Ad5 and CAV-2 vectors in the mouse cornea.** (**a**) Fluorescence detected by *in vivo* microscopy 1-d post-intra-stromal injection of 10^9^ pp of an Ad5 vector expressing EGFP. (**b–d**) EGFP expression is localised to the corneal stroma as determined by histological studies. (**e**) Fluorescence detected by *in vivo* microscopy 1-d post-injection of 10^9^ pp of a CAV-2 vector expressing EGFP. (**f–h**) Histological studies localise EGFP expression to the corneal stroma. Magnifications **a** and **e**: 25×.
(TIF)
