# Supplementary figures and images for: Corneal Transduction by Intra-Stromal Injection of AAV Vectors In Vivo in the Mouse and Ex Vivo in Human Explants
Source: PLoS One. 2012 Apr 16;7(4):e35318. doi: 10.1371/journal.pone.0035318 (PMC3327666; doi:10.1371/journal.pone.0035318)

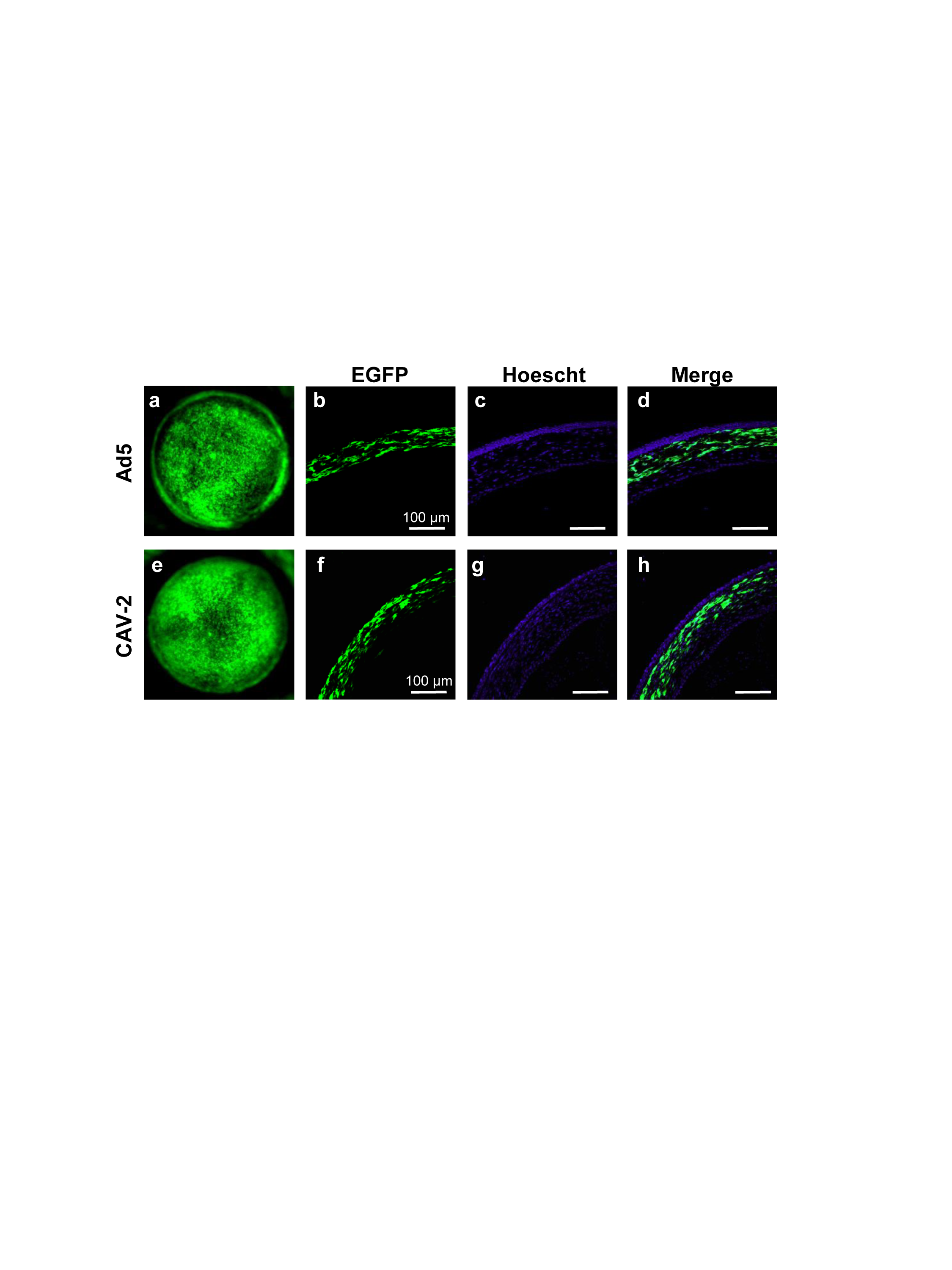

Supplement: Figure S1 — Transduction of Ad5 and CAV-2 vectors in the mouse cornea. (a) Fluorescence detected by in vivo microscopy 1-d post-intra-stromal injection of 109 pp of an Ad5 vector expressing EGFP. (b–d) EGFP expression is localised to the corneal stroma as determined by histological studies. (e) Fluorescence detected by in vivo microscopy 1-d post-injection of 109 pp of a CAV-2 vector expressing EGFP. (f–h) Histological studies localise EGFP expression to the corneal stroma. Magnifications a and e: 25×. (TIF) [file pone.0035318.s001.tif]
